# Supplementary material for: Stray Light in 3D Porous Nanostructures of Single‐Crystalline Copper Film
Source: Small Sci. 2024 Aug 2;4(11):2400174. doi: 10.1002/smsc.202400174 (PMC11934995; doi:10.1002/smsc.202400174)
Supplement: Supplementary file 1 — Supplementary Material [file SMSC-4-2400174-s001.zip › smsc.202400174-sup-0001-suppdata-S1.pdf]

Supporting Information

Stray light in 3D porous nanostructures of single crystalline copper film

Yu-Seong Seo, Teawoo Ha, Ji Hee Yoo, Su Jae Kim, Yousil Lee, Seungje Kim, Young-Hoon Kim, SeungNam Cha, Young-Min Kim, Se-Young Jeong, and Jungseek Hwang**

1. AFM images

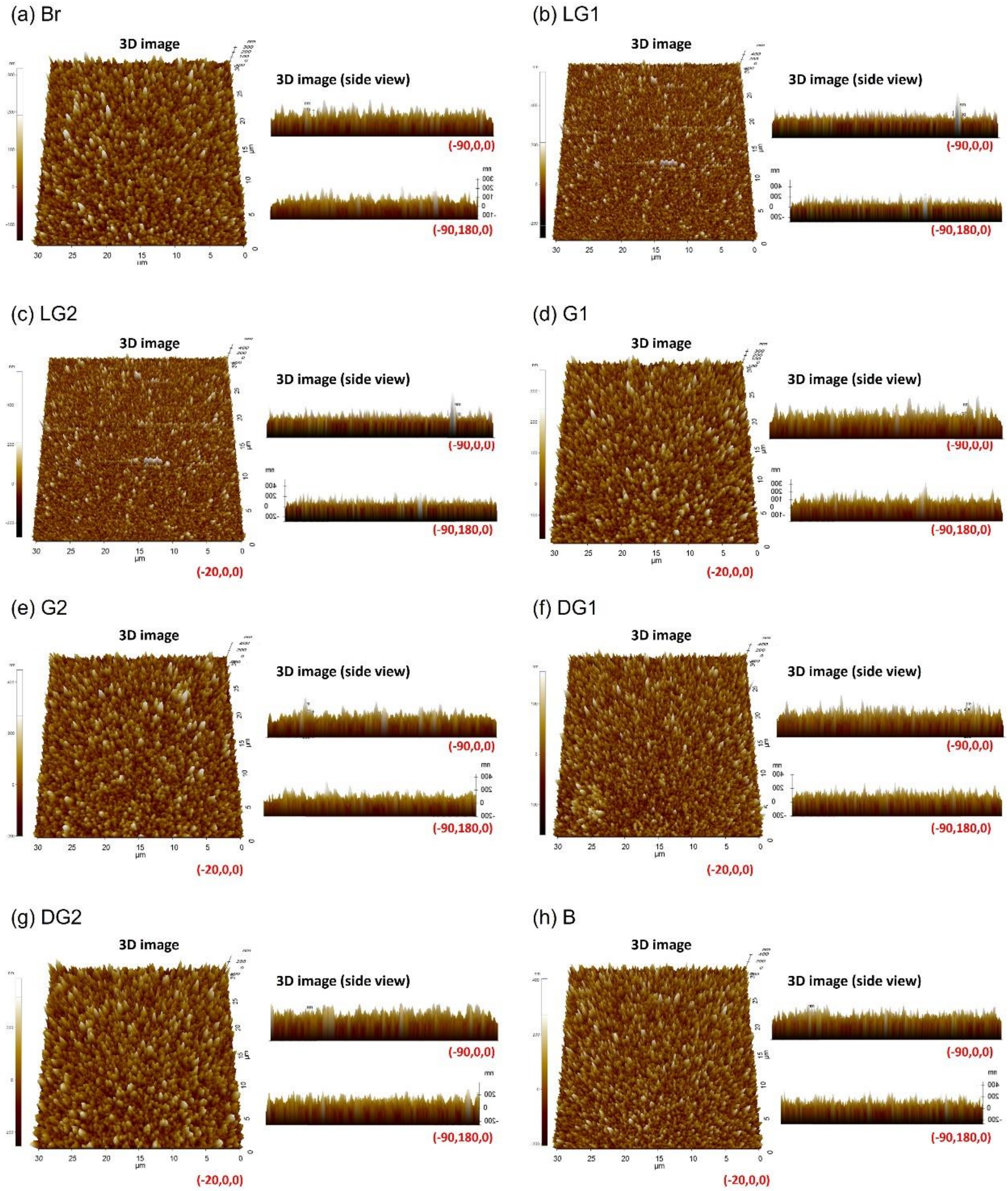

Figure S1. 3D AFM images of all eight ACF samples.

We performed an atomic force microscopy (AFM) study on all eight achromatic copper film (ACF) samples. The resulting images are displayed in Figure S1. The average period of the surface roughness obtained from the power spectral density (PSD) of the AFM image shows a similar sample-dependent trend with the center wavelength estimated from the cutoff in the reflectance spectrum, as shown in Figure 2d. However, its sample-dependent change is smaller.

2. FIB-SEM images

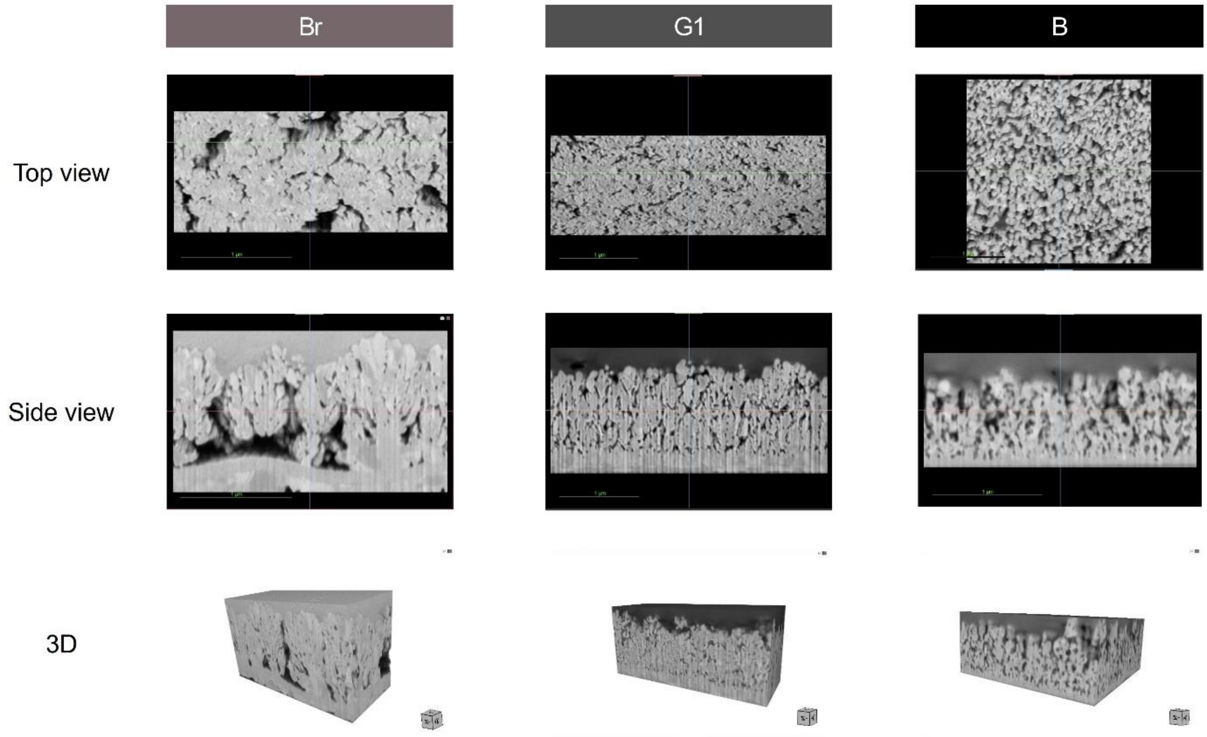

Figure S2. FIB-SEM images of the Br, G1 and B.

Figure S2. displays tomography images of the three samples (Br, G1 and B) from both top and side views, along with a 3D representation. All the images show a noticeable increase in open pores and a decrease in closed pores as the cutoff of the samples decreases. These images support the description of the optical and geometrical properties of the ACF samples mentioned in the main text.

3. FIB-SEM images (open pores)

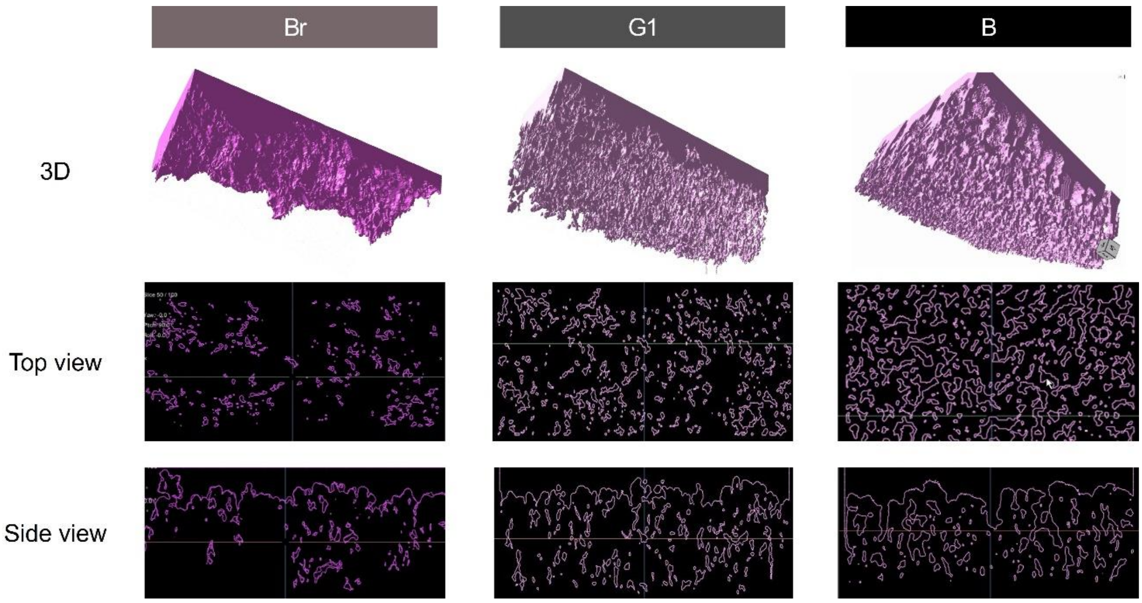

Figure S3. Open pore images of the Br, G1 and B samples.

4. FIB-SEM videos

We have attached video files (file type: ‘*.avi’) from FIB-SEM experiments on three samples (Br, G1 and B). The file names for each sample are as follows:

· Br – ‘ACF_Br_3D.avi’

· G1 – ‘ACF_G1_3D.avi’

· B – ‘ACF_B_3D.avi’
